# Sintering Aid Strategy for Promoting Oxygen Reduction Reaction on High-Performance Double-Layer LaNi_0.6_Fe_0.4_O_3–δ_ Composite Electrode for Devices Based on Solid-State Membranes

**DOI:** 10.3390/membranes13060603

**Published:** 2023-06-15

**Authors:** Denis Osinkin, Nina Bogdanovich

**Affiliations:** 1 Laboratory of Kinetics, Institute of High-Temperature Electrochemistry, Ural Branch of the Russian Academy of Sciences, Yekaterinburg 620066, Russia; bogdanovich@ihte.uran.ru; 2 Department of Life Safety, Institute of Fundamental Education, Ural Federal University, Yekaterinburg 620002, Russia

**Keywords:** LaNi_0.6_Fe_0.4_O_3–δ_, LNF, complex oxide, membranes, sintering aids, electrode, polarization resistance, SOFC, DRT, distribution of relaxation times

## Abstract

Strontium and cobalt-free LaNi_0.6_Fe_0.4_O_3–δ_ is considered one of the most promising electrodes for solid-state electrochemical devices. LaNi_0.6_Fe_0.4_O_3–δ_ has high electrical conductivity, a suitable thermal expansion coefficient, satisfactory tolerance to chromium poisoning, and chemical compatibility with zirconia-based electrolytes. The disadvantage of LaNi_0.6_Fe_0.4_O_3–δ_ is its low oxygen-ion conductivity. In order to increase the oxygen-ion conductivity, a complex oxide based on a doped ceria is added to the LaNi_0.6_Fe_0.4_O_3–δ_. However, this leads to a decrease in the conductivity of the electrode. In this case, a two-layer electrode with a functional composite layer and a collector layer with the addition of sintering additives should be used. In this study, the effect of sintering additives (Bi_0.75_Y_0.25_O_2–δ_ and CuO) in the collector layer on the performance of LaNi_0.6_Fe_0.4_O_3–δ_-based highly active electrodes in contact with the most common solid-state membranes (Zr_0.84_Sc_0.16_O_2–δ_, Ce_0.8_Sm_0.2_O_2–δ_, La_0.85_Sr_0.15_Ga_0.85_Mg_0.15_O_3–δ_, La_10_(SiO_4_)_6_O_3–δ_, and BaCe_0.89_Gd_0.1_Cu_0.01_O_3–δ_) was investigated. It was shown that LaNi_0.6_Fe_0.4_O_3–δ_ has good chemical compatibility with the abovementioned membranes. The best electrochemical activity (polarization resistance about 0.02 Ohm cm^2^ at 800 °C) was obtained for the electrode with 5 wt.% Bi_0.75_Y_0.25_O_1.5_ and 2 wt.% CuO in the collector layer.

## 1. Introduction

Simple solid oxides have attracted much attention due to their diverse physical and chemical properties [1,2,3]. Complex oxides have found numerous applications in high-temperature electrochemical devices for power-to-gas or gas-to-power systems [4,5,6,7]. If the complex oxide has high ionic conductivity, it can be used as an ion-conducting membrane in high-temperature devices [8,9]. If the complex oxide has mixed ionic and electron conductivity, it can be used as an electrode. The most widely studied electrodes to date are various modifications based on (La,Sr)MnO_3±δ_, (La,Sr)CoO_3–δ_ and SrFeO_3–δ_ [10,11,12,13,14]. The common disadvantage of these materials is the presence of strontium, which segregates on the electrode surface at high temperatures. Segregated strontium interacts with carbon dioxide or sulphur-containing impurities to form thermally stable strontium carbonate/sulfate [15,16], leading to electrode degradation. In this context, it is important to investigate strontium-free electrode materials.

Strontium-free LaNi_0.6_Fe_0.4_O_3–δ_ (LNF) is considered one of the most promising materials for oxygen electrodes. LNF has a high electrical conductivity of 100–500 S/cm at operating temperatures [17,18]. The coefficient of the thermal expansion of LNF is approximately 12 × 10^−6^ 1/K [19,20], which is comparable to the most solid-state oxide electrolytes. LNF has a satisfactory tolerance to chromium poisoning [21,22] and is chemically stable to zirconia-based solid electrolytes at electrode-sintering temperatures below 1000 °C [23]. The disadvantage of LNF is its low oxygen-ion conductivity due to the low oxygen vacancy concentration [24]. To increase the oxygen-ion conductivity of the electrode, a complex oxide based on a doped cerium oxide is added to the LNF [25,26,27,28]. For example, in [29] it has been shown that the highest electrochemical activity is exhibited by a composite electrode with an additive based on doped ceria (Ce_1–x_Gd_x_O_2–δ_) in the ratio LNF:Ce_1–x_Gd_x_O_2–δ_ = 1:1. On the other hand, in [30], the electrode with an LNF:Ce_1–x_Gd_x_O_2–δ_ ratio of 7:3 showed the highest activity. It follows that, in any case, the addition of Ce_1–x_Gd_x_O_2–δ_ to the LNF leads to an increase in the electrochemical activity of the electrode. However, it should be noted that the addition of the low-conductivity Ce_1–x_Gd_x_O_2–δ_ reduces the overall conductivity of the composite electrode, which negatively affects the current distribution both in the electrode bulk and at the electrode/current collector interface. In this case, two-layer electrodes with a functional composite layer LNF-doped CeO_2_ and a collector layer of LNF with the addition of sintering additives should be used. The sintering additives are necessary to reduce the sintering temperature of the collector layer so that it is lower than the sintering temperature of the functional layer. It has previously been shown that LNF can be effectively used as a current collector for La_2_NiO_4+δ_ [31]. There are also data on the use of LNF as a collector layer for composite electrodes based on lanthanum manganate [32]. Later, investigations of the Ce_0.8_Sm_0.2_O_2–δ_/LNF-Ce_0.8_Sm_0.2_O_2–δ_/LNF electrode system with the addition of high amounts of sintering additives (Bi_1.6_Er_0.4_O_3–δ_ and Bi_1.5_Y_0.5_O_3–δ_) to the LNF collector layer were published [33,34]. In [33], it was shown that using only YDB in the collector leads to an increase in the electrochemical activity of the bilayer electrode, but does not change the conductivity. Adding only copper oxide to the collector increases the conductivity but does not increase the electrochemical activity of the electrode.

In this study, we optimized the amount of YDB-CuO complex additive, keeping the low temperature electrode formation, high electrical conductivity, and high electrochemical activity. Moreover, in this study, the effect of sintering additives in the current collector layer on the performances of a highly active LNF-based double-layer electrode in contact with the most common solid state membranes (Zr_0.84_Sc_0.16_O_2–δ_, Ce_0.8_Sm_0.2_O_2–δ_, La_0.85_Sr_0.15_Ga_0.85_Mg_0.15_O_3–δ_, La_10_(SiO_4_)_6_O_3–δ_ and BaCe_0.89_Gd_0.1_Cu_0.01_O_3–δ_) was investigated.

## 2. Materials and Methods

### 2.1. Preparation of Dense Electrolyte Substrates

**Figure 1:**
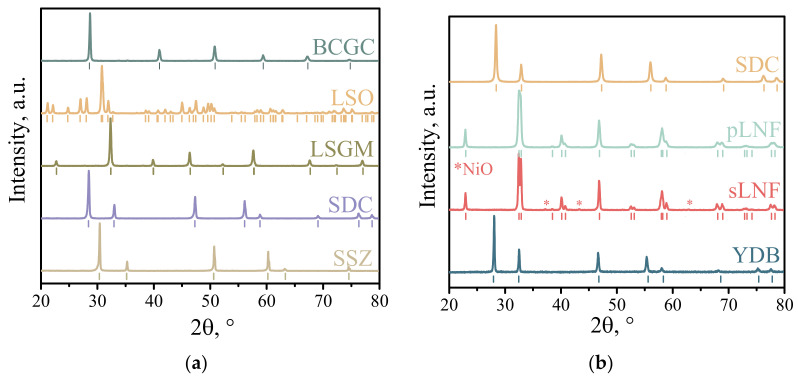
X-ray diffraction patterns for supporting electrolytes (**a**) and powders for the electrodes (**b**).

For the synthesis of La_10_(SiO_4_)_6_O_3–δ_ electrolyte (hereafter LSO) La(NO_3_)_3_·6H_2_O and SiO_2_ were mixed in a planetary mill (Retsch, Haan, Germany) in ethyl alcohol. The required amount of aqueous ammonia solution was added to the mixture under continuous stirring. After drying, the LSO was pre-synthesized at 650 °C for 5 h followed by grinding in a planetary mill. The final synthesis of the LSO was carried out in three stages: in powder at 1100 °C for 3 h followed by grinding, in powder at 1300 °C for 5 h followed by grinding, and in the last stage in pressed tablets at 1600 °C for 5 h. After the final sintering according to XRD data (D/MAX-2200, Rigaku Corporation, Japan diffractometer, Takatsuki, Japan), the LSO was single-phase (Figure 1a) with a relative density of about 93%.

The La_0.85_Sr_0.15_Ga_0.85_Mg_0.15_O_3–δ_ electrolyte (hereafter LSGM) was prepared by the ceramic route using La_2_O_3_, SrCO_3_, MgO, and Ga_2_O_3_ as initial components [35]. The synthesis was carried out in three stages with intermediate grinding of the powder in a PM 100 planetary mill. The intermediate synthesis temperatures were 1100 °C for 2 h and 1150 °C for 5 h. Before the final synthesis, the powders were pressed into tablets and then sintered at 1450 °C for 10 h. After the final synthesis, the LSGM was single-phase according to the XRD data (Figure 1a). The relative density of the LSGM electrolyte was about 97%.

The Ce_0.8_Sm_0.2_O_2–δ_ electrolyte (hereafter SDC) was synthesized by a two-step ceramic method using CeO_2_ and Sm_2_O_3_ by grinding the starting and intermediate products in a planetary mill. The final temperature to produce the dense ceramics was 1550 C for 3 h. According to the XRD data, the SDC electrolyte was single-phase (Figure 1a) with a relative density of about 98%.

The electrolyte Zr_0.84_Sc_0.16_O_2–δ_ (hereafter SSZ) was obtained by hot casting under pressure. The final sintering temperature was 1650 °C for 5 h. According to the XRD data, the SSZ was single-phase (Figure 1a) with a relative density of about 94%.

The BaCe_0.89_Gd_0.1_Cu_0.01_O_3–δ_ proton-conducting electrolyte (hereafter BCGC) with a final synthesis temperature of 1400 °C for 5 h was obtained by the solid phase method according to the procedure described in [36]. According to the XRD data, the BCGC was single-phase (Figure 1a) with a relative density of about 96%.

### 2.2. Synthesis of Powders for Electrodes

The powder LaNi_0.6_Fe_0.4_O_3–δ_ (hereafter LNF) was synthesized by two methods: the Pechini method and the solid phase method. The synthesis of LNF by the Pechini method (pLNF) is described in detail in [33,34]. The final synthesis temperature was 900 °C for 6 h. XRD showed that the synthesized pLNF powder was single-phase (Figure 1b). The specific surface area of the obtained pLNF powder was 5.5 m^2^/g (SORBI N4.1, META, Novosibirsk, Russia). For the synthesis of LNF by the solid-state method (sLNF), La_2_O_3_, Fe_2_O_3_, and NiO were used as initial components. The starting components were mixed in a planetary mill in the required proportions. The preliminary synthesis was carried out at 1150 °C for 2 h. The final synthesis was carried out in two stages at 1250 °C for 5 h and 1270 °C for 5 h with an intermediate grinding of the powders in the mill. XRD showed that the powder contained a second phase, nickel oxide, the content of which did not exceed 2 wt.% (Figure 1b). The specific surface area of the synthesized sLNF powder was 1.6 m^2^/g.

The SDC electrolyte powder for the functional electrode layer was prepared by combustion of a solution containing Ce(NO_3_)_3_, Sm(NO_3_)_3_, and glycine. The solution was evaporated to form a xerogel in which a redox reaction was carried out under further heating. After synthesis, the powder was ground in a ball mill. Heat treatment was carried out at 700 °C for 8 h and at 1100 °C for 8 h. After the final synthesis, a single-phase powder was obtained (Figure 1b). The specific surface area of the powder was 12.2 m^2^/g.

The synthesis of the sintering additive Bi_0.75_Y_0.25_O_1.5_ (hereafter YDB) for the collector layer of the electrode was carried out by the nitrate combustion method. The method is described in detail in [33]. The XRD results showed that the obtained YDB material was single-phase (Figure 1b). The specific surface area of the powder was 1.85 m^2^/g. Highly dispersed copper oxide powder was prepared by electric explosion of copper wire in an oxygen atmosphere [37]. The specific surface area of the powder was 10.3 m^2^/g.

### 2.3. Sample Preparation

In this study, five types of symmetrical electrochemical cells with bilayer electrodes were investigated. The functional electrode layer was the same in all cases. For the functional layer, pLNF powder obtained by the Pechini method was mixed with electrolyte powder SDC in a 1:1 mass ratio with the addition of alcohol and polyvinyl butyral without using sintering aids. The electrode slurry of the functional layer was applied to the surfaces of SSZ, SDC, LSGM, LSO, and BCGC electrolytes and sintered at 1000 °C for 2 h. The thickness of the functional layer after sintering was in the range of 15–17 µm in all cases.

Electrode slurries for the electrode collector layer were prepared by mixing the pLNF, sLNF, YDB, and CuO powders in the required proportions with ethyl alcohol and polyvinyl butyral binder. The collector layer was applied to the functional layer of the electrode and sintered at 900 °C for 2 h. The total thickness of the bilayer electrodes after sintering was in the range of 36–44 µm. The compositions of the collector layers and their designations are given in Table 1.

To investigate the electrical conductivity of the collector layers, samples with an SDC- supporting electrolyte were prepared in the form of an elongated plate. Electrode slurry was applied to one surface of the plate and fired at 900 °C for 2 h.

The SEM images of the electrodes and energy-dispersive X-ray analysis were performed using a VEGA (Tescan, Brno, Czech Republic) electron microscope with an INCA Energy 350 energy-dispersive X-ray microanalysis system (Oxford Instruments, Abingdon, UK).

### 2.4. High-Temperature Measurements

Electrical conductivity measurements were carried out by the conventional 4-probe DC method using a 2700 multimeter (Keithley Instruments, Solon, OH, USA). Current and potential probes were made of platinum wire and fixed on platinum paste at 850 °C for 30 min. The electrochemical performance of the electrodes of electrochemical cells was studied by means of impedance spectroscopy using a Solartron FRA-1260 and EI-1287 (Ametek, Hampshire, UK). The experimental setup is described in [38]. The distribution of relaxation time (DRT) method was used to analyze the impedance data. The DRT analysis was performed using the program code developed by the authors of [39] based on Tikhonov’s regularization.

## 3. Results

### 3.1. Chemical Compatibility

**Figure 2:**
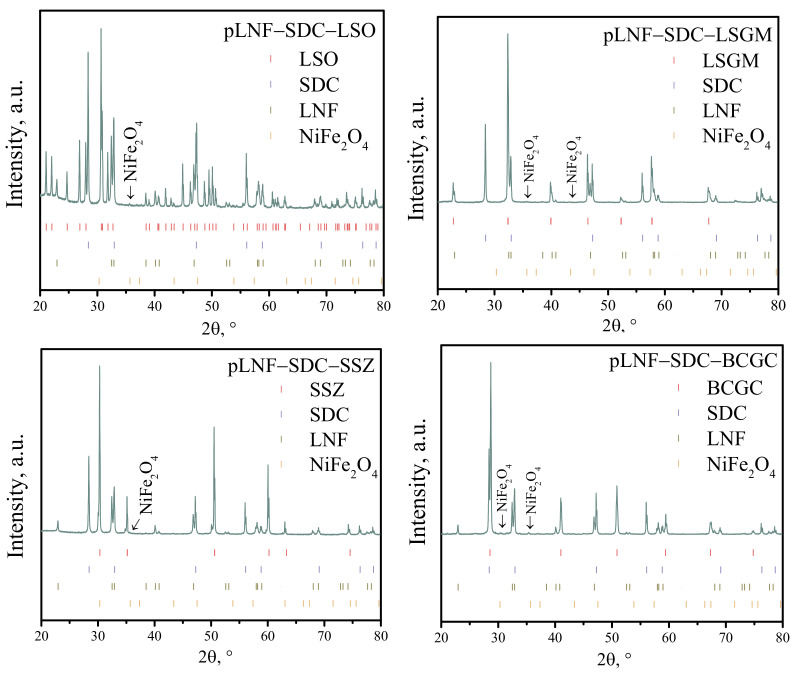
X-ray diffraction patterns of pLNF-SDC-electrolyte powders after firing at 1000 °C for 5 h in air.

In the first stage of the study, the chemical compatibility between the functional electrode layer and the supporting electrolytes was investigated. For this purpose, the pLNF-SDC composite powder was mixed in a mass ratio of 1:1 with the powder, which was obtained by grinding a dense tablet of the supporting electrolyte. The pLNF-SDC-electrolyte powder was then annealed at 1000 °C for 5 h in an air atmosphere. X-ray patterns of the pLNF-SDC-electrolyte powders are shown in Figure 2. The chemical compatibility of the collector layer with the electrolyte was not investigated because in electrochemical cells the collector layer is not in contact with the supporting electrolyte but only with the functional layer.

As can be seen in Figure 2 for the annealed pLNF-SDC mixture with LSO, LSGM, BCGC, and SSZ powders, in addition to the peaks for the main phases, there is a reflex from the NiFe_2_O_4_ phase, the main reflex of which is at 35.7 degrees. The intensity of this peak indicates that the mass fraction of NiFe_2_O_4_ in the composite powder does not exceed 2 wt.%. Considering that the NiFe_2_O_4_ phase does not contain any cations from the electrolyte, it can be said that the chemical compatibility of all electrolytes with the functional layer of the cathode is satisfactory. Apparently, the formation of NiFe_2_O_4_ is due to the fact that the annealing of the composite powder was carried out at a higher temperature than in the synthesis of the pLNF. It is worth noting that in [40] a peak at 35.7° was also detected for LNF obtained by the Pechini method at an annealing temperature of 1000 °C.

### 3.2. Electrical Conductivity of Collector Layers

**Figure 3:**
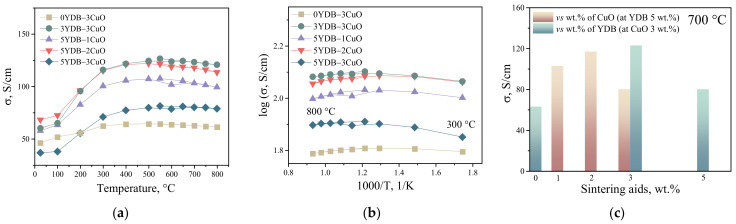
Electrical conductivity of collector porous layers with different contents of sintering additives. The composition of the collector layers is shown in Table 1. In linear coordinates (**a**), in Arrhenius plot (**b**) and depending on the type and content of the sintering additive (**c**).

The results of the electrical conductivity study of collector layers with sintering additives are shown in Figure 3. As can be seen, the conductivity of the collector layer depends on the amount and nature of the sintering additive and varies in the range of 60 to 120 S/cm at 700 °C. The sample with only copper oxide as sintering additive showed the lowest conductivity values. The sample with 3 wt.% copper oxide and 3 wt.% YDB showed the best results. A similar result was shown in [33], i.e., the use of two sintering additives at once instead of one leads to a more positive effect. As stated in [33,34], the introduction of YDB and CuO additives in the collector layer results in an expansion of both the contact area between the electrode particles and the area of their contact with the functional layer. Up to a certain point, this leads to a better electron transport in the cathode and the extension of the reaction area into the electrode bulk. A low dependence of the conductivity in the temperature range 300–800 °C is observed for all the investigated compositions. The low activation energy of conductivity is characteristic of LNF due to the metallic type of conductivity [17,18]. It is problematic to compare the obtained values of electrical conductivity of collector layers with the literature, because in most of the papers the electrical conductivity is studied on samples obtained by pressing with subsequent sintering at high temperatures, i.e., these are samples with high density. In our case, the collector layer for electrical conductivity studies was produced by a method similar to that used to produce it on the surface of the functional layer of the electrochemical cell. In other words, the collector layer is thin and has a high porosity, about 45%, so that the conductivity values shown in Figure 3 are lower compared to the literature data [17,18].

**Figure 4:**
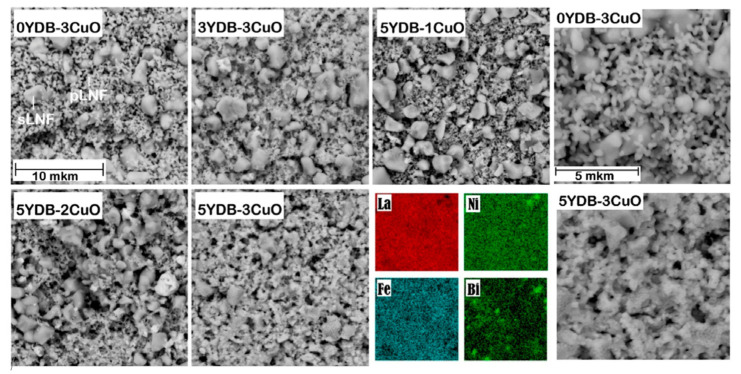
SEM images of collector layers with different proportions of sintering aids and maps of elements distribution.

The SEM images of collector layer surfaces with different proportions of sintering aids fraction are shown in Figure 4. As can be seen, two types of particles are clearly visible in all cases: large particles of about 1 µm (is sLNF) and small particles whose size is difficult to determine (is pLNF). With increasing sintering additives in the electrode, the size and distribution of the sLNF particles remain almost unchanged. However, the pLNF particles change significantly. For example, in the 0YDB–3CuO electrode, the boundaries between the pLNF particles are clearly visible, whereas in the 5YDB–3CuO electrode the pLNF particles are agglomerated, which seems to result in a higher electrical conductivity of the collector layers with two sintering additives. Elemental analysis using the EDX method showed an almost uniform distribution of elements in the electrode (Figure 4).

### 3.3. Electrochemical Activity

**Figure 5:**
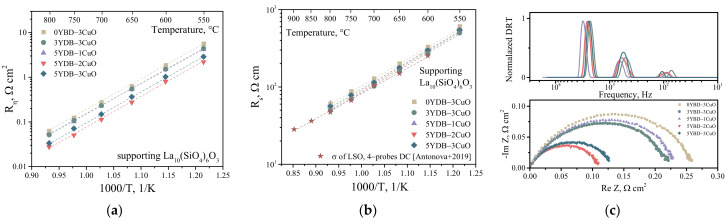
Temperature dependences of polarization resistance of bilayer electrodes in contact with LSO electrolyte (**a**), temperature dependences of series resistance of electrochemical cells, the bottom dependence is taken from [41] (**b**), EIS spectra and DRT functions for 700 °C (**c**).

The electrochemical activity of the electrodes was studied on symmetrical cells with bilayer electrodes and supporting electrolyte. The functional layer was the same for all the cells: 50 wt.% pLNF + 50 wt.% SDC. The compositions of the collector layers and their designations are given in Table 1. In the first stage of the electrochemical activity study, five types of bilayer electrodes were investigated in contact with the La_10_(SiO_4_)_6_O_3–δ_ (LSO) electrolyte, Figure 5a.

As can be seen, the obtained temperature dependence of the polarization resistance for all cells shows a linear behaviour in the investigated temperature range. The bilayer electrode with 5YDB–2CuO collector layer showed the best electrochemical activity. The value of polarization resistance at 800 °C was about 0.02 Ohm cm^2^. Although the 3YDB–3CuO collector layer showed the highest electrical conductivity (Figure 3), the electrochemical activity of the electrode with the 3YDB–3CuO collector layer did not show the best results. This seems to be due to the different mechanism of conductive contacts formation on the surfaces of the dense electrolyte SDC (in the case of layer resistance measurement) and the porous functional layer. When analyzing the ohmic resistance of electrochemical cells, it was found that the ohmic resistance of the cell increases with increasing sintering additives in the collector layer of the electrode, Figure 5b. As the conductivity of the collector layer increases, the ohmic resistance of the cell becomes comparable to the ohmic resistance of the LSO electrolyte as measured by the 4-probe DC method [41]. This clearly indicates that the use of a highly conductive collector layer also affects the uniformity of the current distribution at the electrode/electrolyte interface.

The electrochemical impedance spectra of cells with LSO-supporting electrolyte and bilayer electrodes with different amounts of sintering additives are shown in Figure 5c. As can be seen, the spectra represent one semicircle. Depending on the concentration of sintering additive in the collector layer of the electrode, the appearance of the impedance spectrum does not change, only the polarization resistance does. In [42], the authors also noted an increase in electrode activity without a change in the nature of the impedance spectrum when different current collectors were used. The DRT functions calculated from these impedance spectra are shown in Figure 5c. It can be seen that there are three peaks in the DRT function corresponding to the three stages of the oxygen reduction reaction. The three stages of the electrode reaction on similar electrodes have been shown previously [34] and were related to charge transfer, bulk diffusion, and surface phenomena. The most important information is that the electrode reaction mechanism does not change depending on the concentration of sintering additives in the current collector, as the intensity and frequency of the relaxation peaks remain unchanged.

**Figure 6:**
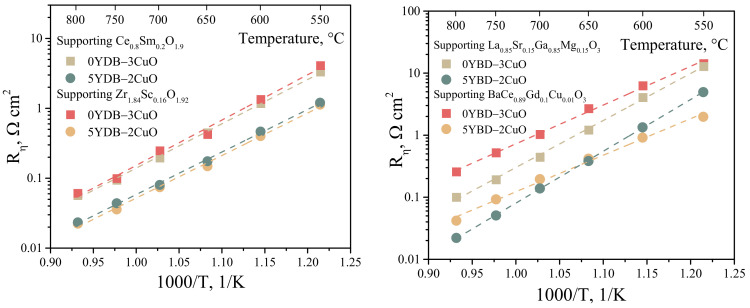
Temperature dependence of the polarization resistance of bilayer electrodes in contact with different electrolytes.

The electrochemical cells with SDC-, LSGM-, BCGC-, and SSZ-supporting electrolytes were further investigated. The collectors 0YDB–3CuO (as the most inefficient) and 5YDB–2CuO (as the most efficient) were chosen on the basis of the data shown in Figure 5a. The results of the polarization resistance of the bilayer electrodes in contact with different electrolytes are shown in Figure 6. It can be seen that for all types of supporting electrolytes, the electrochemical activity of the electrode increases approximately two to five times with a more conductive collector layer (Table 2). The values of polarization resistance for cells with a 5YDB–2CuO layer were about 0.02–0.04 Ohm cm^2^ at 800 °C for all supporting electrolytes, which is an excellent result compared to active electrodes (Table 3). In summary, for all supporting electrolytes, there is a significant improvement in the electrochemical activity of the composite electrode with the use of a highly conductive collector layer.

**Figure 7:**
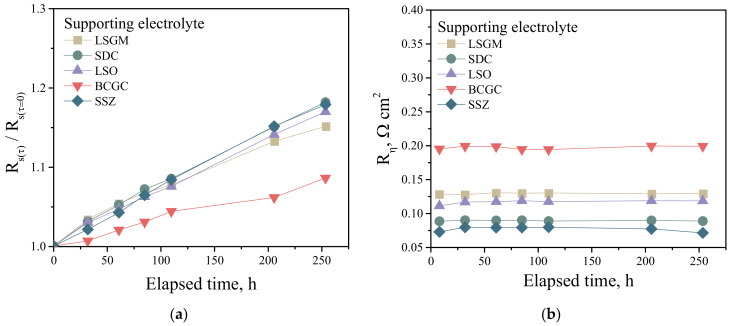
Time dependence of the relative series resistance of electrochemical cells and (**a**) polarization resistance of a double layer electrode with 5YDB–2CuO collector layer for (**b**) cells with different supporting electrolytes.

In order to determine the stability of the electrochemical performance of the electrodes over time, electrochemical cells with different supporting electrolytes and with the 5YDB–2CuO collector layer were tested at 700 °C for 250 h. Figure 7a shows the time dependence of the relative series resistance of the cells. As can be seen, the resistance varies over the time studied. Since the series resistance of electrochemical cells is mainly determined by two parameters, namely the ohmic resistance of the supporting electrolyte and the contact area of the electrode, but changes in these two components can influence the observed behaviour of the series resistance.

The change in the ohmic resistance of the electrolyte can be attributed to the ordering of defects in the crystalline structure of the electrolyte at high temperatures, which leads to a reduction in the conductivity of the material. The high porosity of the electrode in high-temperature electrochemical cells makes it susceptible to sintering [33], with a reduction in the contact area with the supporting electrolyte and consequently an increase in the series resistance of the cell and the polarization resistance of the electrode. However, as shown in Figure 7b, the polarization resistance of the electrodes remains almost unchanged for 250 h. In [33] it was noted that the LNF-based double layer electrodes have a low reduction in electrochemical activity over time when collector layers with several sintering additives are used. It can therefore be said that the observed change in series resistance over time is due to a decrease in the conductivity of the supporting electrolyte.

## 4. Conclusions

This study presents the results of the investigation of the effect of sintering additives (Bi_0.75_Y_0.25_O_2–δ_ and CuO) in the current collector layer on the performance of highly active LaNi_0.6_Fe_0.4_O_3–δ_-based electrodes in contact with the most common solid-state membranes: Zr_0.84_Sc0_.16_O_2–δ_, Ce_0.8_Sm_0.2_O_2–δ_, La_0.85_Sr_0.15_Ga_0.85_Mg_0.15_O_3–δ_, La_10_(SiO_4_)_6_O_3–δ_, and BaCe_0.89_Gd_0.1_Cu_0.01_O_3–δ_. It was established that LaNi_0.6_Fe_0.4_O_3–δ_ have good chemical compatibility with the above membranes at a co-sintering temperature of 1000 °C. It has been shown that the conductivity of the electrode collector layer depends on the amount and nature of the sintering additive and varies in the range of 60 to 120 S/cm at 700 °C. The sample with only copper oxide as a sintering aid showed the lowest conductivity values. The sample with 3 wt.% copper oxide and 3 wt.% Bi_0.75_Y_0.25_O_2–δ_ showed the best results for electrical conductivity. The electrochemical activity of the bilayer electrode with an SDC-LNF functional layer enhances as the conductivity of the collector layer increases, due to a more uniform current distribution. It was shown that the mechanism of the oxygen reduction reaction does not change depending on the type and concentration of sintering additives in the collector layer of the electrode. It was found that electrodes with sintering additives 5 wt.% Bi_0.75_Y_0.25_O_2–δ_ and 2 wt.% copper oxide in the collector layer had the highest electrochemical activity. The two-layer electrode with high performance collector layer was shown to be highly stable for 250 h at 700 °C.

## Figures and Tables

**Table 1:** Composition of the collector layers and their designations.

Composition of the Collector Layer/wt.%	Designations
60% sLNF + 32% pLNF + 5% YDB + 3% CuO	5YDB–3CuO
60% sLNF + 33% pLNF + 5% YDB + 2% CuO	5YDB–2CuO
60% sLNF + 34% pLNF + 5% YDB + 1% CuO	5YDB–1CuO
60% sLNF + 34% pLNF + 3% YDB + 3% CuO	3YDB–3CuO
60% sLNF + 37% pLNF + 3% CuO	0YDB–3CuO

**Table 2:** Polarization resistance of bilayer electrodes in contact with different electrolytes.

Sample	Supporting Electrolyte and Polarization Resistance (Ohm cm^2^) at 800/700/600 °C				
	LSO	LSGM	SDC	SSZ	BCGC
0YDB–3CuO	0.06/0.28/1.83	0.09/0.45/3.75	0.05/0.19/1.11	0.06/0.25/1.36	0.25/1.03/6.15
5YDB–2CuO	0.03/0.11/0.79	0.02/0.13/1.27	0.02/0.08/0.47	0.02/0.07/0.39	0.04/0.19/0.89

**Table 3:** Comparison of polarization resistance with literature data.

Composition	Polarization Resistance (Ohm cm^2^)	Reference
YBaCo2O_5–δ_	0.13 (700 °C)	[43]
La_2_NiO_4+δ_ + Pr_2_NiO_4+δ_	0.15 (700 °C)	[44]
Sr_2_Fe_1.5_Mo_0.5_O_6–δ_	0.14 (800 °C)	[45]
YBa_0.8_Sr_0.2_Co_2_O_5-δ_	0.20 (700 °C)	[46]
La_0.6_Sr_0.4_Fe_0.9_Sc_0.1_O_3-δ_	0.015 (800 °C)	[47]
PrBaMn_2_O_5-δ_	0.30 (800 °C)	[48]
La_0.4_Sr_0.6_Co_0.2_Fe_0.7_Nb_0.1_O_3–δ_ + GDC	0.034 (800 °C)	[49]
NdBaCo_2/3_Fe_2/3_Cu_2/3_O_5+δ_	0.05 (800 °C)	[50]
CaMn_0.95_P_0.05_O_3−_*_δ_*	0.3 (800 °C)	[51]
Pr_1.7_Ba_0.3_NiO_4+δ_	0.4 (700 °C)	[52]

## Data Availability

Not applicable.
